# Does artificial intelligence need companionship to assist in drug discovery? The Kirsten rat sarcoma virus study

**DOI:** 10.1093/bjrai/ubae001

**Published:** 2024-03-04

**Authors:** Mourad Stitou, John M Koomen, Denis J Imbody, Yi Liao, Andrii Monastyrskyi, Uwe Rix, Derek R Duckett, Eric B Haura, Aleksandra Karolak

**Affiliations:** Department of Machine Learning, Moffitt Cancer Center, Tampa, FL 33612, United States; Department of Molecular Oncology, Moffitt Cancer Center, Tampa, FL 33612, United States; Department of Thoracic Oncology, Moffitt Cancer Center, Tampa, FL 33612, United States; Department of Drug Discovery, Moffitt Cancer Center, Tampa, FL 33612, United States; Department of Drug Discovery, Moffitt Cancer Center, Tampa, FL 33612, United States; Department of Drug Discovery, Moffitt Cancer Center, Tampa, FL 33612, United States; Department of Drug Discovery, Moffitt Cancer Center, Tampa, FL 33612, United States; Department of Thoracic Oncology, Moffitt Cancer Center, Tampa, FL 33612, United States; Department of Machine Learning, Moffitt Cancer Center, Tampa, FL 33612, United States

**Keywords:** AI, drug discovery, fragment-based drug discovery, acquired resistance

## Abstract

In this *Opinion* article, we confront the role of artificial intelligence (AI) in targeting and understanding resistance to targeted therapy using the most frequently mutated oncoprotein family in human cancer, rat sarcoma virus guanosine triphosphate hydrolases (RAS GTPases), here Kirsten RAS (KRAS), as an example. Aberrant regulation of the active GTP-bound state of KRAS is associated with tumourigenesis, aggressive disease, and poor prognosis. KRAS mutations (eg, G12C, G12D, G12V, G13D, inter al.) are drivers of numerous cancer types, including non-small cell lung, colorectal, and pancreatic cancers. These mutations have shown to play a significant role in cell behaviour and response to treatment. Since its discovery in the 1980s, it has been recognized that over-expression of KRAS and other RAS family members induces resistance to radiotherapy. Moreover, over the years preclinical and clinical studies showed that tumours with KRAS mutations exhibit different treatment sensitivities compared to tumours with wild-type KRAS.

## The conventional wisdom of undruggable KRAS

Until this decade, KRAS was considered undruggable.^1^^,^^2^ The featureless character of KRAS at the molecular level including smooth and spherical shape and lack of evident binding sites or pockets stalled many research initiatives. KRAS acts as guanine diphosphate/triphosphate GDP/GTP switch responsible for signal transduction between activated receptors on the membrane and cellular components.^3^ GTP binding induces conformational change that triggers KRAS activation, while KRAS mutations lock the mutant in its active GTP-bound state.^4^ Therefore, higher cellular concentrations of GTP over GDP, higher (picomolar) affinity of KRAS towards GTP, and altered off-rates of KRAS mutants towards GTP increased the ‘drugging challenge’.^3–5^

## The promising prospects

With the development of KRAS G12C small molecule irreversible inhibitors, overcoming these challenges became a reality.^6^ In 2021, the Food and Drug Administration (FDA) approved AMG510 (sotorasib),^7^ an allele-specific covalent inhibitor of the KRAS G12C mutant. MRTX849 (adagrasib, approved in 2022^8^) displayed clinical responses across multiple tumour types and showed promising activity against cancers harbouring this KRAS mutation. Unfortunately, tumours with KRAS mutations eventually develop resistance to monotherapy following either ‘on-target’ or ‘off-target’ resistance mechanisms. The ‘on-target’ resistance is characterized by secondary KRAS mutations affecting binding of the inhibitor, while other isoforms of KRAS or MAPK signalling pathway members become activated in the ‘off-target’ resistance.^4^ Several KRAS secondary mutations might interfere with key protein-drug interactions (eg, Y96D, Y96C) and trigger resistance to KRAS G12C inhibitors.^1^^,^^9^ Even though a distinct KRAS G12C active-state inhibitor, RM-018, demonstrated the ability to overcome resistance by targeting KRAS G12C/Y96D double mutation in the binding sites^10^ (other tricomplex inhibitors targeting KRAS G12C are now in clinical trials^11^), such development of novel mutations following treatment or radiotherapy presents an even more formidable challenge.

To address the challenges posed by resistance to first-generation KRAS monotherapy by acquired KRAS mutations, researchers have been actively exploring various possibilities and therapies that could advance KRAS targeting either through development of optimized drugs or radiation. Current leading strategies to sensitize tumours to radiation and enhancement of treatment efficacy focus on combined therapies targeting signalling pathways of mutated KRAS;^12^ however, it remains unclear whether the combination therapies will work in patients.

## Call for cross-disciplinary collaborations

In our opinion, understanding the mechanisms and dynamics of specific KRAS mutations is the key to (i) guide treatment decisions in chemotherapy, targeted therapy, and radiation oncology, (ii) predict secondary mutational processes that may appear, and (iii) develop strategies to overcome the resulting drug resistance. However, no progress can be made without cross-disciplinary collaborative initiatives that integrate various scientific perspectives, including structural biology, functional genomics, and pharmacology. Despite constraints of limited data and bottlenecks of high-throughput screening (HTS)^13^ (eg, target validation, candidate optimization, and constraints in experimental testing), recent artificial intelligence (AI)-based studies present promising opportunities to transform drug discovery^14^ with several examples reaching clinical testing (NCT05154240 and published studies^15^^,^^16^). AI techniques can learn and self-correct, improving their performance with each new dataset. As AI technologies gain experience, the quality of the data used becomes crucial, adhering to the principle of ‘garbage in, garbage out' and the paradox of acquiring large sample size versus high-quality data.^16^ This challenge impacts their ability to enhance accuracy and making them powerful tools to analyse data to evaluate complex clinical scenarios, like secondary resistance to KRAS inhibitors in patients’ tumours.

Artificial intelligence-guided drug discovery may encounter failures due to various reasons, including the lack of sufficient high-quality data and the black-box nature of certain AI models (especially observed for neural network architectures of multiple hidden layers, high dimensionality of the features, and utilization of nonlinear functions), hindering interpretability or understanding of their predictions, and limiting their acceptance in critical decision-making processes. To overcome these challenges, researchers should prioritize building comprehensive databases and repositories to capture emerging mutations and therapeutic responses. Data sharing initiatives and open-access repositories could create a vast and accessible pool of data to fuel AI-guided drug discovery. Additionally, development of specialized AI models capable of handling the complexities of drug-protein interactions and the complex pathways is essential. AI architectures, such as graph-based models, show potential to better interpret complex biological data and analyse complex biological processes that could address drug resistance. Furthermore, to be successful, drug discovery requires integration of AI model strengths with experimental HTS data. In our opinion, by verifying AI-guided predictions through experiments and iteratively improving AI techniques based on experimental feedback, researchers can further optimize and automate drug discovery. Overall, through interdisciplinary collaboration, innovative methodologies, and an emphasis on interpretability and validation, AI can become an invaluable tool in drug discovery.^17^

In conjunction with experimental efforts, genomic analyses, histologic evaluations, and molecular simulations, AI holds tremendous promise in overcoming drug resistance in KRAS mutant cancers. Here, we provide our perspective on top key strategies incorporating AI, which can create successful novel therapeutic strategies:

- ***Integration of AI with Genomic and Imaging Data***: Recent studies utilizing genomic and histologic analyses have shed light on the diverse mechanisms of acquired resistance to covalent KRAS G12C inhibitors. These mechanisms can include acquired KRAS alterations, bypassing signalling pathways, and phenotypic or histologic transformations (eg, epithelial-to-mesenchymal transition as well as small cell lung cancer or squamous cell lung cancer transformation). While many insights have been gained into therapeutic evasion, there is still much to learn about the full spectrum of resistance mechanisms and their clinical implications. Several challenges must be addressed to fully realize AI benefits in overcoming resistance to drugs in KRAS mutants. The strategic use of AI as a crucial tool for integrating and standardizing diverse datasets, specifically genomic and imaging data, is essential, eliminating many of the challenges that researchers face, leaving only experimental validation to be done. Additionally, the dynamic nature of resistance necessitates continuous adaptation of AI and other models to keep pace with evolving resistance mechanisms.
- ***Fragment-Based Drug Discovery and High-Throughput Screening coupled with AI*:** Fragment-Based Drug Discovery (FBDD)^13^ holds great potential for enhancing the treatment of tumours with cancer-driving mutations.^18^ The recent approval of sotorasib marked a breakthrough in the success of FBDD.^19^ However, the appearance of acquired resistance that challenges direct KRAS inhibition is exacerbated by incomplete understanding how mutation affects the binding pocket. Moreover, traditional FBDD approaches encounter limitations that undermine the effectiveness in addressing complex drug targets. These limitations include diversity and size of the fragment libraries, overlaps and uniqueness among commercially available fragments, and issues related to solubility and stability that can be compounded when combining fragments. As AI approaches emerge, they carry potential to address these limitations and enhance FBDD by enabling the cost-efficient design of fragment libraries, hit optimization, and acceleration of lead compound identification. The concept of AI-derived protein-specific fragment libraries and automated fragment enhancers presents a promising topic. ‘Deconstruction’ of existing drugs or effective linking and growing of fragments to generate compound libraries (current challenge of FBDD) are examples that could be accelerated by AI-enabled examination of binding affinity, selectivity, and synthetic accessibility.^20^ By training AI on HTS results and transfer learning to resistant targets, AI-supported FBDD could offer a powerful approach to propose new KRAS inhibitors that overcome drug resistance caused by secondary mutations.
- ***Integration of Structural Modelling, Molecular Simulations, and AI***: Molecular docking and molecular dynamics simulations play a vital role in predicting drug-protein interactions and understanding the dynamic movement between predicted drugs and protein binding sites. Covalent docking enables the identification of potential initial molecules that can form covalent bonds with the target site of KRAS G12C or double mutants. Standard or quantum mechanics molecular simulations can provide data and insights into the understudied conformational changes of binding site due to acquired mutations,^9^ stability and conformational changes of drug-protein complexes, and aid the AI-enabled design of next-generation optimized inhibitors.^21^ To understand resistance arising from the changes in the protein, AI can integrate structural modelling, docking, and molecular dynamics simulation with pharmacophore modelling;^13^^,^^22^^,^^23^ strategies that can lead to novel targeted therapies against resistance-causing mutations and improved treatment outcomes. This powerful combination enables the evaluation of drug-protein interactions and provides crucial insights into the binding modes upon treatment-acquired mutations.

## Potential of human and machine intelligence

In summary, AI can serve as a bridge, facilitating interdisciplinary research by integrating expertise from medicinal chemistry, molecular and computational biology, bioinformatics, and pharmacology.^22^^,^^23^ The synergy of these fields, combined with AI-driven analyses, allows for a comprehensive understanding of resistance mechanisms and the development of effective therapeutic strategies and translation to the clinic.^16^ The collaborative efforts between AI, other computational, and experimental research are crucial for translating these discoveries into clinical applications and advancing personalized medicine approaches for patients in need.^24^ Further identification of specific biomarkers associated with KRAS mutations may help predict mutations and tumour response to therapy, enabling clinicians to select the most effective treatment strategy. We believe that only through collaborative efforts and communication across disciplines we can unlock the full potential of human and machine intelligence against oncogenic mutations, leading to personalized and effective treatments for patients with resistant tumours.
